# Direct Transamidation Reactions: Mechanism and Recent Advances

**DOI:** 10.3390/molecules23092382

**Published:** 2018-09-18

**Authors:** Paola Acosta-Guzmán, Alejandra Mateus-Gómez, Diego Gamba-Sánchez

**Affiliations:** Laboratory of Organic Synthesis Bio- and Organocatalysis, Chemistry Department, Universidad de los Andes, Cra. 1 No 18A-12 Q:305, Bogotá 111711, Colombia; pa.acostag@uniandes.edu.co (P.A.-G.); a.mateus@uniandes.edu.co (A.M.-G.)

**Keywords:** transamidation, amide, amine, catalyst, catalysis

## Abstract

Amides are undeniably some of the most important compounds in Nature and the chemical industry, being present in biomolecules, materials, pharmaceuticals and many other substances. Unfortunately, the traditional synthesis of amides suffers from some important drawbacks, principally the use of stoichiometric activators or the need to use highly reactive carboxylic acid derivatives. In recent years, the transamidation reaction has emerged as a valuable alternative to prepare amides. The reactivity of amides makes their direct reaction with nitrogen nucleophiles difficult; thus, the direct transamidation reaction needs a catalyst in order to activate the amide moiety and to promote the completion of the reaction because equilibrium is established. In this review, we present research on direct transamidation reactions ranging from studies of the mechanism to the recent developments of more applicable and versatile methodologies, emphasizing those reactions involving activation with metal catalysts.

## 1. Introduction

The amide functionality has been recognized as one of the most important functional groups, not only because of its widespread presence in Nature (in proteins, peptides, and alkaloids, among others) [1] but also because of the vast number of synthetic structures bearing this group [2]. It is estimated that approximately 25% of the existing pharmaceuticals contain an amide bond as part of their structures [3] and that approximately 33% of the new drug candidates are “amides” [4], thus making amidation reactions some of the most performed chemical processes in the pharmaceutical industry and in drug discovery activities [5].

Traditional methods to synthesize amides suffer from significant issues, principally the use of stoichiometric amounts of activating reagents with the consequent production of waste or the use of corrosive and troublesome reagents such as acyl chlorides or anhydrides. As a consequence, the ACS Green Chemistry Institute assessed the amide bond formation with good atom economy as one of the biggest challenges for organic chemists [6]. In recent years, amide bond synthesis by nontraditional methods has been reviewed, and some alternatives are available to perform acylations on nitrogen [7,8,9,10]. Among those unconventional methods, the transamidation reaction appears to be a useful strategy.

The acyl exchange between an amide and an amine has been known since 1876 with the studies carried out by Flescher [11]; however, the method was pretty limited. For approximately one hundred years, the transamidation reaction was almost unexplored, and only a few successful examples were published [12,13,14,15]. The biggest drawback of this method was the use of high temperatures and very long reaction times. The first catalytic transamidation was performed using carbon dioxide [14]; however, the quantity used makes it not properly a catalyst but rather a promoter; additionally, the yields were always lower than 67%. It was not until 1994 that a modern and complete study on a direct transamidation was published by Bertrand and coworkers [16]. Their work was based on the use of aluminum chloride as a promoter, and the method was limited to the use of aliphatic amines since low yields were obtained with aromatic amides. Chemists noticed the importance and the potential of the direct transamidation reaction and developed some useful and general methods performed under the influence of different types of catalysts. Today, heterogeneous, metallic, acidic and basic catalysts are available to perform transamidation reactions. In this review article, we will begin with the mechanistic studies for transamidation of primary and secondary amides, followed by studies of the reaction with tertiary amides and some other mechanistic studies using proline as a catalyst. In the following sections, we will present a set of selected examples of direct transamidation reactions catalyzed by metals as well as some alternative catalysts.

## 2. Mechanistic Studies

In transamidation, an alternative strategy to prepare amides, the direct exchange of the amine moiety in an amide can occur only under the influence of a suitable catalytic or stoichiometric activating agent because of the poor electrophilic nature of amides. Depending on the reaction conditions, the structure of the amide, and the activating agent employed, the reactivity of these amides differs from reaction to reaction; however, it follows the regular pattern that the primary amides are more active than the secondary and tertiary amides. Nevertheless, some activating agents capable of activating secondary amides have been described in the literature with considerable success [10]. The order of reactivity shown before implies that the structure of the amide is the most important constraint in transamidation processes. Another very important factor is the acidic nature of the N-H function, which could hamper the process by simply inactivating the catalytic species. Considering these facts, understanding how this process occurs represents one of the main challenges for the organic chemistry community [8].

**Scheme 1:**
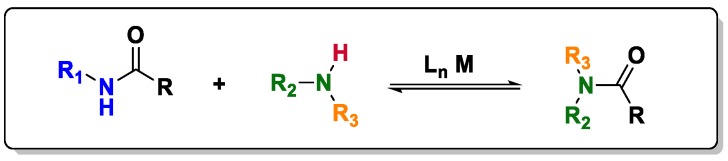
Transamidation of a secondary amide with a secondary amine.

In this sense, Stahl and coworkers [17,18,19] studied the mechanism of the transamidation reaction catalyzed by metals, focusing on the reaction between a secondary amine and a secondary amide (Scheme 1). They evaluated the behavior of nucleophilic alkali-metal amides, Lewis acidic metal complexes, transition metals and different amides. The authors found that metallic complexes of titanium and aluminum could catalyze the transamidation reaction due to their relatively low basicity; furthermore, metallic complexes of titanium [Ti(NMe_2_)_4_] and aluminum [Al(NMe_2_)_6_] could catalyze the transamidation reaction due to an increase in electron density in the metal center. This effect is related to the reduced basicity of the ligand, which increases the Lewis acidic character of the metal.

**Scheme 2:**
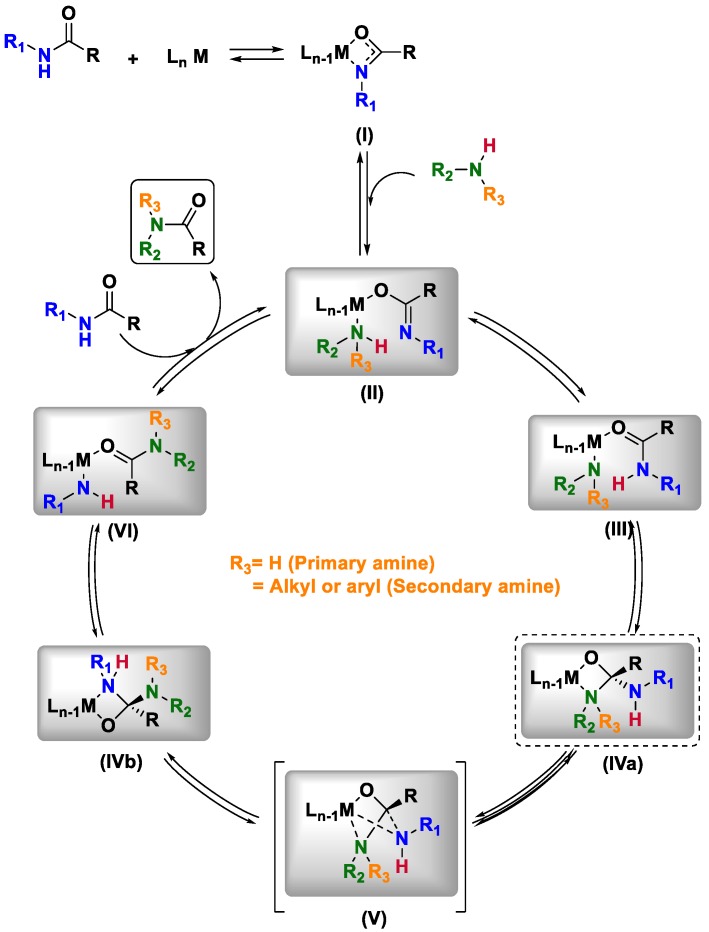
Proposed catalytic cycle for transamidation reactions between primary or secondary amides with primary amines.

Based on the previous information, the author carried out a complete mechanistic study, which included the determination of the deuterium kinetic isotope effect, the rate law and the identity of the catalyst resting state. With the results obtained in the kinetic studies, they proposed a catalytic mechanism to explain the transamidation process (Scheme 2). They suggested that in the first step, the secondary amide reacts with the precatalyst to yield metal-amidate complex **I**. Once this complex is formed, it enters the catalytic cycle by a bimolecular reaction with a primary amine to generate adduct **II**. After that, a proton transfer between the nitrogen atoms is proposed to reach intermediate **III**. Later, an intramolecular nucleophilic attack of the amido-ligand to the carbonyl of the amide forms metallacycle **IVa**. This intermediate is the most important species in the catalytic cycle because it embodies the bifunctional role of the metal center *via* activation of both the amine and amide substrate. However, this intermediate can either revert the cycle to complex **III** or continue the catalytic cycle by isomerization to form **IVb**. The isomerization proceeds by transition state **V**, where the metal is linked to both nitrogen atoms, thus changing the nitrogen coordinated to the metal center and providing exchanged intermediate **IVb**. This product is easily converted into **VI**, and finally, the breakdown of intermediate **VI** promoted by a second molecule of amide leads to the formation of the desired amide and the liberation of an amine molecule that is responsible, in most of the cases, for the equilibrium displacements; in other words: it is the driving force of this reaction [18,19,20].

**Scheme 3:**
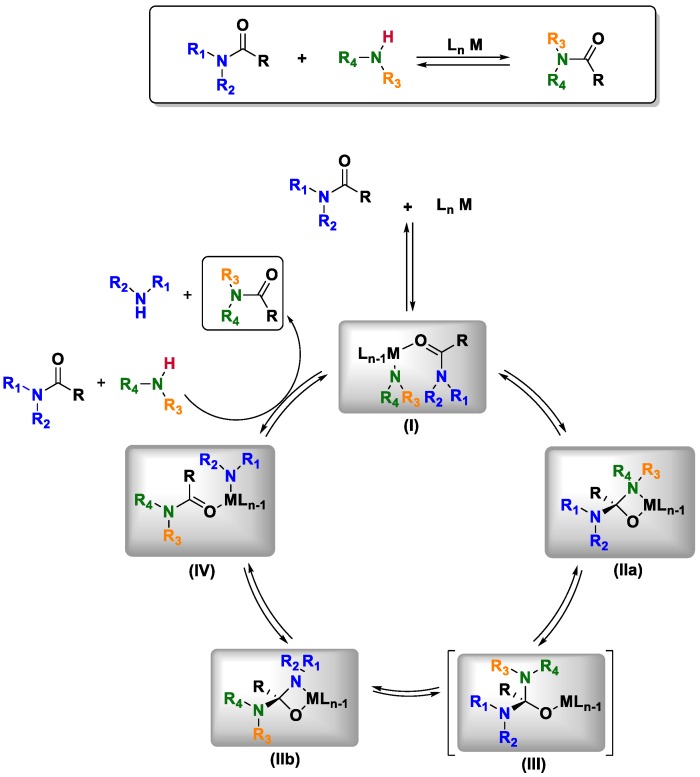
Proposed catalytic cycle for transamidation reaction between tertiary amides with secondary amines.

Careful analysis of the proposed mechanism suggests that tertiary amides are unsuitable reagents for the transamidation reaction. This is because of the absence of an N-H bond and the consequently different reactivity between these amides and bases. However, successful examples of transamidation reactions with tertiary amides have been described in the literature, suggesting that tertiary amides should follow a different mechanistic pathway. Stahl and coworkers confronted this issue and developed a new study focused on the mechanistic details of the transamidation reaction between tertiary amides and secondary amines employing kinetic, spectroscopic, and computational studies (Scheme 3). According to their results, the first step occurs when the amide interacts with the metal complex to form intermediate **I**, where the simultaneous activation of the electrophile (amide) and the nucleophile (amine) by the metal center is clear. After that, an intramolecular attack of the amido ligand to the coordinated amide gives rise to metal-stabilized tetrahedral intermediate **IIa**, which can interconvert to its isomeric intermediate **IIb** by a simple cleavage and coordination sequence through intermediate III. In the last step, a new amine/amido pair coordinated to the metal **IV** is formed, from which it is possible to obtain the desired product [21,22].

Comparing the transamidation processes for secondary amides and tertiary amides makes some similarities evident. First, the mechanisms exhibit not only that there are obvious structural similarity between the intermediates but also that the key step in both processes is the intramolecular nucleophilic attack of the amido ligand on a metal-coordinated amide. Regarding the differences between the two transamidation processes, perhaps the most important one is the identity of the catalyst resting state, implying substantial differences in the kinetic properties of both reactions since the transamidation reaction of secondary amides follows first-order kinetics with regards to the metal and amine involved and the amide follows zero-order kinetics. Contrary to that, for the tertiary amide, the rate law is zero-order for the amine, is half- to first-order for the metal and varies from zero-order to saturation behavior for the amide. The transamidation of primary amides is easier than the cases described before because ammonia is produced in the reaction medium, thus making the reaction simpler and usually better yielding. Concerning the mechanisms, each research team describes its own proposal, typically based on the Stahl studies. In our opinion, slight differences between metals should exist, and as a consequence, the reaction mechanism for the transamidation of primary amides should be pretty similar to that described for secondary amides. Additionally, the large number of non-metal-catalyzed transamidation reactions makes the investigation of their mechanisms interesting. Unfortunately, only one computational study on the reaction catalyzed by l-proline has been described; mechanisms with other catalysts remain unexplored.

The organocatalyzed transamidation reaction has been described as a greener methodology compared with the metal-catalyzed version of this reaction. l-Proline has received much attention due to its dual role as a ligand and a catalyst in other reactions. In view of the above perceptions, l-proline was described as a useful catalyst for the transamidation reaction. Adimurthy and coworkers [23] found that various amides react with a variety of amines in the presence of l-proline as a catalyst. The reaction scope included benzylamines with electron-rich and deficient substituents and alkyl aromatic, aliphatic, and secondary amines, which reacted with remarkable ease. Specifically, the reactions of benzylamine with primary amides showed good yields compared to reactions with secondary and tertiary amides. The authors also demonstrated that the reactions have a high degree of functional group tolerance. The mechanistic study carried out to explain the role of l-proline and to disclose why the transamidation can be efficiently catalyzed by proline was reported by Xue and coworkers some years after the reaction was described. They used density functional theory (DFT) calculations to achieve a reasonable proposal.

**Scheme 4:**
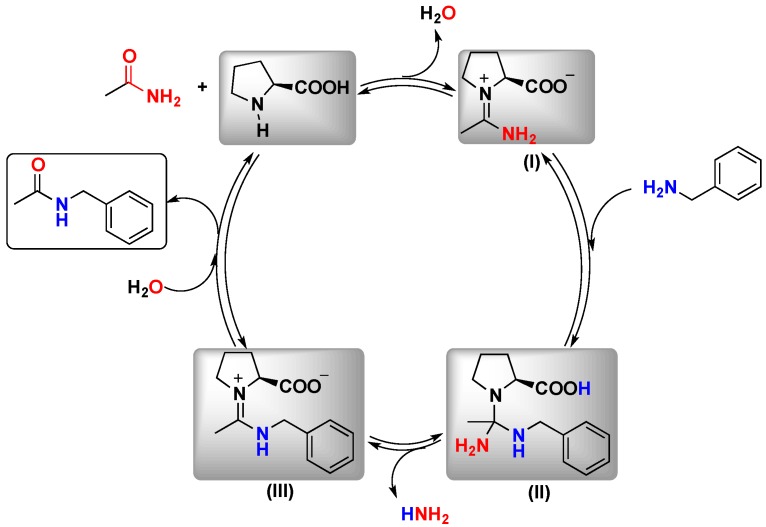
Proposed mechanism to explain the transamidation reaction catalyzed by l-proline.

The transamidation of acetamide with benzylamine was selected as the model system. The calculations revealed that the reactions catalyzed by l-proline follow a stepwise mechanism, which involves a first step where the activation of the amide takes place through a proton transfer from l-proline to the carbonyl group of the amide to form intermediate imidine **I**. Subsequently, a nucleophilic addition of benzylamine to the imidine intermediate occurs to form **II**. After eliminating one molecule of ammonia, a hydrolysis of intermediate **III** produces the target product. This study allowed the researchers to identify the hydrolysis reaction as the rate-determining step (RDS) in the catalytic cycle with the largest energy barrier (26.0, 26.8 and 29.7 kcal/mol in toluene, ethanol and H_2_O respectively) (Scheme 4) [24].

As already mentioned, the mechanistic studies on the transamidation reaction are quite limited; however, the metal-catalyzed version presumably follows similar mechanisms independent of the metal identity, with the mechanistic pathway depending on the type of amide reacting. Concerning the non-metal-catalyzed version, some examples are described in the literature, but among them, only l-proline has received enough attention that a mechanistic study was carried out. Nevertheless, the summary of mechanistic studies presented in this section is sufficient to understand the principles and particularities of transamidation reactions. In the following chapters, we will present some selected examples of transamidations and discuss their particularities and issues.

## 3. Metal-Catalyzed Direct Transamidations

**Scheme 5:**
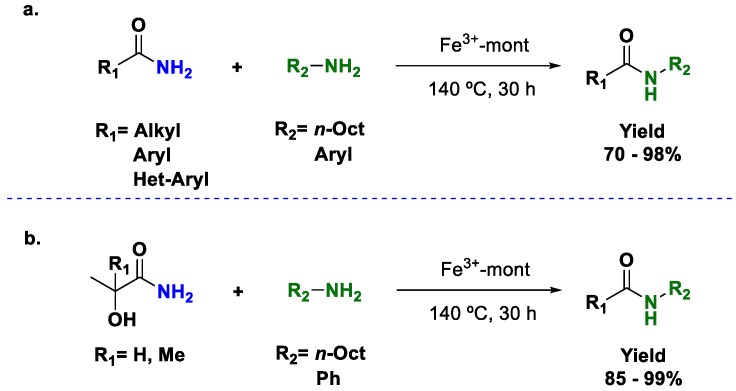
(**a**) Transamidation of amides (alkyl, aryl, Het-aryl) with amines catalyzed by Fe^3+^-mont. (**b**) Transamidations of α-hydroxyamides with amines.

Since the pioneering work reported by Stahl and coworkers [18,19,20,21,22], several metal catalysts have been described for direct transamidation reactions. Among them, one of the most commonly used is iron(III), particularly in the form of simple Fe(III) salts, supported in clay or even as nanoparticles. The first study was reported in 2014 by Shimizu and coworkers [25]; they used Fe(III) supported in montmorillonite to perform the direct transamidation of primary amides (Scheme 5). The authors showed that the reaction can be performed under solvent-free conditions with a slight excess of amine at 140 °C.

**Scheme 6:**
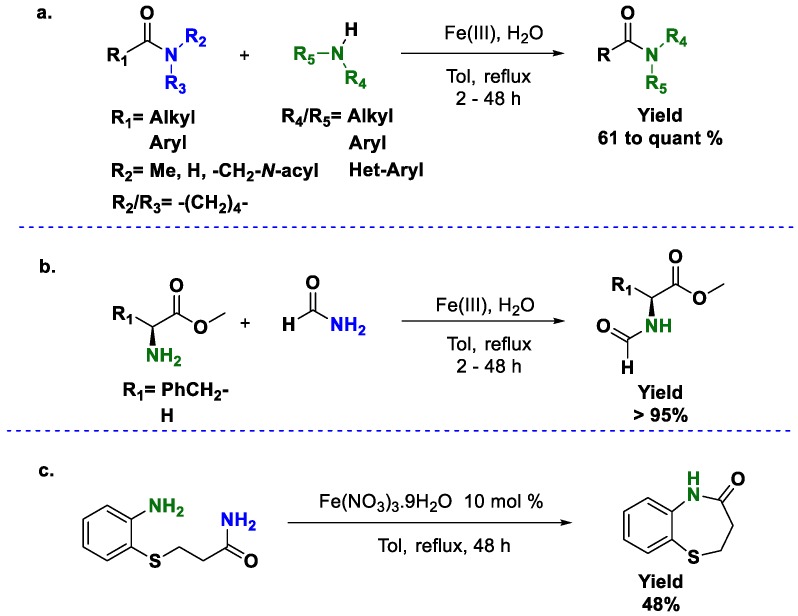
Transamidation catalyzed by Fe(III) and water of (**a**) carboxamides. (**b**) α-amino esters. (**c**) Synthesis of 2,3-dihydro-*5H*-benzo[*b*]-1,4-thiazepin-4-one by intramolecular transamidation.

Unfortunately, the scope of the reaction is limited; only primary amides and primary amines can be used (Scheme 5a). Additionally, the method is limited to the use of liquid starting materials since at least one of the two reagents has to be a liquid, thus making this method unsuitable for a large number of reagents. A few examples with free α-hydroxy amides were described (Scheme 5b). Typically, reactions carried out under solvent-free conditions are seen as good examples of green chemistry principles; however, the transamidation reaction has to be seen from another perspective. As we mentioned before, amidation reactions are one of the most performed processes in drug discovery and the pharmaceutical industry, and the structure of amides used in those fields are somehow complex, meaning they are usually polyfunctional compounds with more than 20 carbon atoms or, in some cases, amino acid derivatives. As a consequence, a good method to synthesize amides has to be amenable to all the structures mentioned before, and solid reagents are highly desirable even if the use of a solvent becomes imperative. This has served us as inspiration to develop a more general method for direct transamidations using Fe(III) [26]. Our method is based on the use of simple Fe(III) salts. In fact, we demonstrated that any Fe(III) salt can be used as a catalyst for this reaction; the sole condition is the presence of water, which has a complementary catalytic role in this process. Water can be added or obtained from hydrated salts, the identity of which is only limited by the availability of the reagents. Technical grade toluene is also a suitable solvent. Consequently, this method is one of the cheapest reported in the literature (Scheme 6).

The reaction scope is very good since the reaction can be performed not only with an excess of amides or amines but also with stoichiometric quantities. The reaction time depends on the amide used, but it is usually shorter with primary amides and longer with secondary and tertiary amides. We also described the transamidation of secondary amides where the liberated amine is not a gas or a volatile liquid and showed that Fe(III)/water is a suitable catalyst in those cases. It should be noted that no previous functionalization of the secondary amide was needed (Scheme 6a). Finally, the reaction was described with some amino acids as the amino source (Scheme 6b), and one intramolecular example was provided (Scheme 6c). A few years later, magnetic nanoparticles were used in direct transamidation reactions, with some advantages and issues. The method described by Thale [27] using Fe_3_O_4_ has the same problem that all transamidations performed under solvent-free conditions show. The loading of the catalyst is also high (20 wt.%), and only primary amines are used as nucleophiles. However, this particular method has some advantages: the catalyst is easily recovered and can be used in several reactions without loss of activity. It is also simple to prepare, and the reaction can be performed with DMF as an example of a tertiary amide. On the other hand, the method described by Heidari and Arefi [28] has a better substrate scope and uses smaller quantities of catalyst. Unfortunately, the preparation process for the catalyst is more complicated and uses simple iron salts as precursors; this is potentially problematic since the authors did not quantify the free salts in their catalyst, and it is possible that some remaining salts are responsible for the transformation, on the other hand if the simple salts are good and suitable catalysts for this reactions, prepare a catalysts that do not provide very different results using them a starting materials consumes time unnecessarily. The recovery of the catalyst was also described, but the identity of the catalytic species is still unclear; both Heidari and Thale attribute the catalytic activity to the metal center. 

**Scheme 7:**
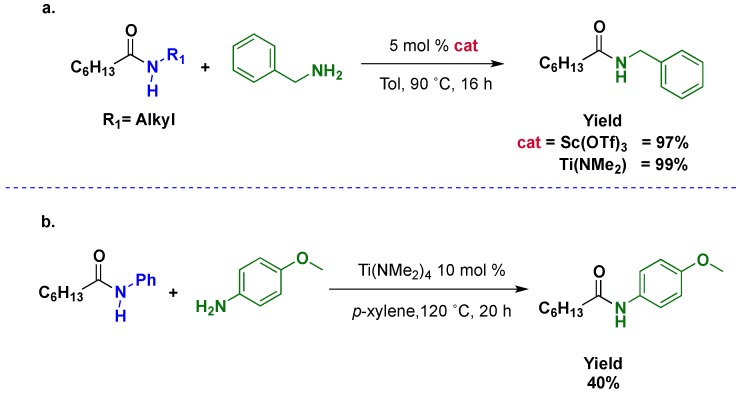
Catalytic transamidation of (**a**) *N*-alkyl heptylamines with benzylamine and (**b**) *N*-aryl amides with aryl amines.

It is clear that the transamidation reaction is an equilibrium, and as such, when ammonia or gaseous amines are liberated in the reaction media, the reaction is easier. On the other hand, when the liberated amine is a liquid, the reaction becomes more complicated. Stahl and Gellman et al. [29] faced this problem and developed a method applicable to *N*-aryl amides. In this case, Ti(NMe_2_)_4_ and Sc(OTf)_3_ were suitable catalysts affording good yields and acceptable selectivities to the cross-product. The reaction was performed with nonpolar solvents and at low temperatures. However, the best results were obtained with an excess of aliphatic amines (Scheme 7a); the use of stoichiometric aromatic amines produced mixtures of products (Scheme 7b).

The reactions described before have some common characteristics: they are all performed in nonpolar solvents, and an excess (at least 1 equiv.) of one of the reagents is needed to obtain complete conversions and good yields. One interesting development was the use of Cu(OAc)_2_ as a catalyst described by Beller and coworkers [30], who used *tert*-amyl alcohol as the solvent and 0.3 excess equivalents of amine. The reaction is limited to the use of primary amides but has a very good substrate scope, as it tolerates free OH groups in the substrate and can be performed with chiral reagents (amides or amines) without racemization. The sole problem is the use of sealed tubes since high pressures are a safety issue in organic chemistry laboratories.

**Scheme 8:**
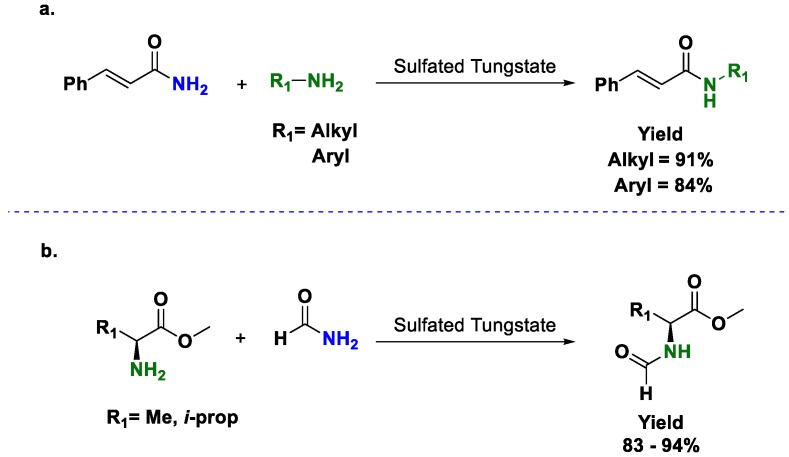
Transamidation reactions in the presence of sulfated tungstate of (**a**) cinnamide with alkyl and aryl amines and (**b**) α-amino esters with formamide.

As the reader can notice, several efforts have been made to have more active and general catalysts for transamidations. Simple metal salts have been proven as suitable catalysts for this transformation, however, the use of more developed metal catalysts may have some advantages. In particular, the use of sulfated tungstate proved to be effective with α,β-unsaturated amides [31]. These substrates can be potentially complicated because of the presence of two reactive sites. Once the amide forms the complex with the metal center, the carbonyl is more electrophilic, but the β position suffers a similar activation; as a consequence, Michael additions are competitive reactions. In the case of sulfated tungstate, no Michael product was observed (Scheme 8a). The catalyst was also active with α-amino acid esters, as observed in Scheme 8b.

**Scheme 9:**
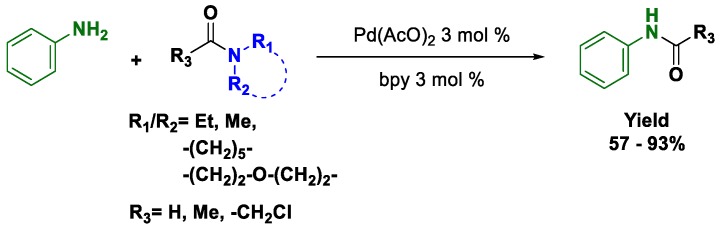
Transamidation of DMF derivatives with aniline.

The use of tertiary amides is typically exemplified with DMF, and very few examples with other tertiary amides have been described. Fortunately, the use of Pd(OAc)_2_ with 2,2′-bipyridine (bpy) as the ligand can be used for the transamidation of tertiary amides [32]. This catalyst afforded very good yields with aromatic amines, which are easily oxidized and consequently gave lower yields than their aliphatic partners (Scheme 9). The biggest issue of this method is the use of a large excess of amine: 10 equivalents are required. However, the reaction can be performed on a gram scale with excellent results. 

Very recently, Hu and coworkers [33] described a reductive transamidation of tertiary amides (very challenging substrates) with nitrobenzenes promoted by manganese. Presumably, the manganese acts not only as a reducing agent by producing the amine in situ but also as an activating agent for the amide. This was proven by adding some metals usually found as impurities of manganese, such as palladium or nickel. Additionally, this metal has also been described as a useful catalyst in a new method of transamidations, such as a reaction where the amide is activated by the use of BOC or tosyl groups to increase their reactivity. There are some amazing recent publications in this field, but this are not within the scope of this review article. The reaction performed by Hu *et al*. showed very good substrate scope and, to the best of our knowledge, is the sole general method for direct transamidation of tertiary amides.

From the previous examples, it is clear that the transamidation reaction is a powerful alternative in amide synthesis. The use of metal catalysts has shown tremendous growth in the last few years, and many researchers have developed a vast variety of catalysts using metallic centers. We selected the most general and simplest methods to discuss in this review article. Nevertheless, other less important catalysts have to be superficially mentioned, including manganese oxide (MnO_2_), which was used under solvent-free conditions [34] with a limited substrate scope but with good yields. The use of lanthanides has also been described; in particular, a bimetallic lanthanum alkoxide [35], and immobilized Ce(III) [36] were used as heterogeneous catalysts with the associated advantages, such as easy catalyst recovery and low catalyst charge, but also with the normal issues, such as the catalyst preparation and activation. Some more innovative catalysts have been prepared and used successfully in transamidation. Mesoporous niobium oxide spheres [37] and *N*-heterocyclic carbene ruthenium (II) complexes [38] are some of the most general and easy to use catalysts with the best substrate scope. Very recently, the use of Co(AcO)_2_⋅4H_2_O was described [39] with an excellent substrate scope, but there are no special considerations to mention here.

Some of the catalysts presented in this section can be used with ureas, with phthalimide and, in very few cases, with thioureas. The activation of those substrates has proven more difficult than amides, even though the C–N bond has similar features to them. In addition, no details are provided about the application of transamidation reactions with ureas and phthalimides since it is outside the scope of this review. Concerning the catalysts, the development of nonmetallic catalysts has received comparable attention and will be discussed in the next chapter.

## 4. Other Catalysts

**Scheme 10:**
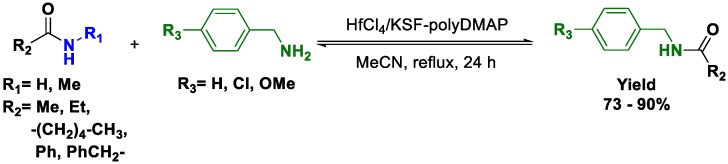
Transamidation reaction catalyzed by HfCl_4_/KSF-polyDMAP.

In an effort to develop safe, environmentally benign, economical, and energy saving chemical reactions, the design and synthesis of recyclable catalysts have become an important objective. In this context, innovative catalysts for transamidations have been developed. One of the most active catalysts is the recyclable polymer-based metallic Lewis acid catalyst developed by Cui and coworkers [40]. This catalyst, which is based on HfCl_4_/KSF-polyDMAP, is recyclable and was described for transamidations of different amide/amine pairs (Scheme 10). The authors chose HfCl_4_ because it is active in the direct condensation of carboxylic acids and alcohols and because the Hf(IV) salts are easily manipulated and applicable in large-scale reactions. During the optimization process, the authors tried an HF/KSF system and found it suitable for transamidations with mainly nonpolar or low polarity solvents. Interestingly, this study led them to discover that by using mixtures of solvents such as acetonitrile/water and acetonitrile/NH_3_, the yield was increased. The use of HfCl_4_/KSF with polyDMAP or NH_3_ afforded even better yields; presumably, the reaction rate is increased as a result the change in Lewis acidity when polyDMAP and NH_3_ coordinate to the metal center (Hf). This catalytic system was applied to a variety of aliphatic amides and benzylamines with excellent results. Additionally, the HfCl_4_/KSF-polyDMA catalyst can be easily recovered and reused in the same reaction at least five times without loss of activity. However, the catalytic system has some limitations when other aliphatic amines are used. 

The use of stoichiometric boron reagents for amidation reactions has also been reported several times in the literature [8]. Borate esters were used by Sheppard et al. [41] to activate amides in transamidations. They evaluated different boron reagents and found that B(OMe)_3_ and B(OCH_2_CF_3_)_3_ show the best behavior in carboxamidation and transamidation processes. This B(OCH_2_CF_3_)_3_-mediated transamidation reaction gave secondary amides in good yields; unfortunately, the main focus of this study was direct carboxamidation, and consequently, very few examples of transamidations were provided. A big issue in this is the use of stoichiometric amounts of the boron reagent. Consequently, the development of new methods that use catalytic amounts of boron reagents is highly desirable. 

**Scheme 11:**
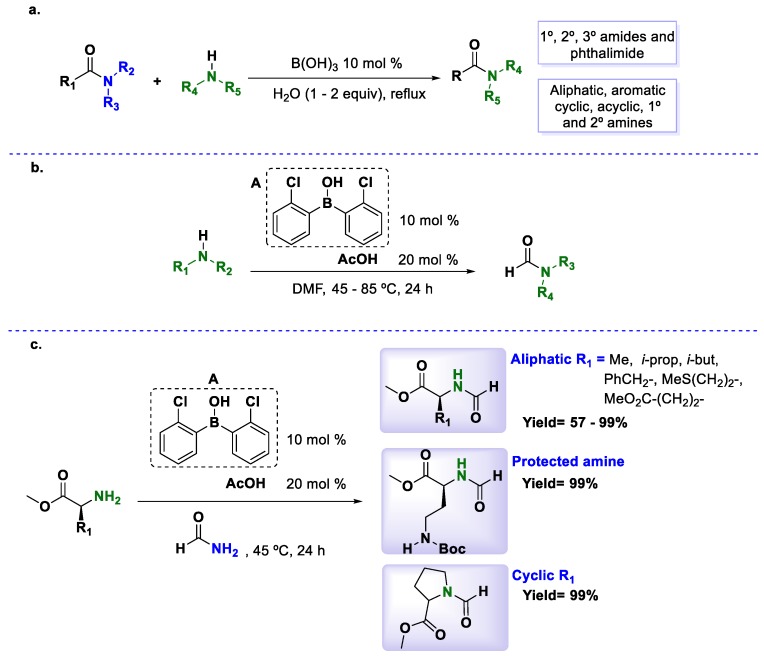
(**a**) Boron-mediated transamidation of primary, secondary and tertiary amides with different kinds of amines. (**b**) Formylation of amines catalyzed by a boronic acid. (**c**) Transamidation catalyzed by boron between α-amino esters and formamide.

Nguyen et al. [42] developed the first transamidation using substoichiometric amounts of boron derivatives. In that research, the authors studied boric acid-catalyzed transamidations of amides and phthalimide. Different solvents were tested in an attempt to increase the reaction conversion. As is typical for this kind of reaction, nonpolar solvents such as toluene or xylene showed good results; however, a higher conversion was observed when the reaction was performed under solvent-free conditions. Additionally, the presence of water (1–2 equivalent) helped promote the transamidation process. Different substrates were used successfully and good yields were obtained in most cases. Secondary amines such as morpholine or piperidine proved to be less reactive and higher temperatures were needed in order to obtain acceptable yields. The most important advantage of this method compared with the other reports is the applicability on primary, secondary and tertiary amides (Scheme 11a). 

However, the high temperatures needed to carry out this method have limited the reaction to using stable achiral amides. To circumvent this problem, Blanchet and coworker [43] exploited the remarkable efficiency of boronic acids as catalysts for the transamidation of DMF with amines under lower temperatures (Scheme 11b). Many tests were carried out, and the results demonstrated that the combination of 10 mol % of boronic acid **A** and 20 mol % of acetic acid was the best combination to promote the transamidation reactions (Scheme 11b). The authors proposed a cooperation between both acids and that a Lewis acid-assisted Bronsted acid (LBA) catalytic system could be involved. Excellent yields were found in all cases, and the process showed very good behavior with primary and secondary amines as well as α-amino esters. Nonetheless, to perform the reaction with chiral substrates, the DMF solvent was changed to formamide in order to keep racemization at a low level (Scheme 11c).

**Scheme 12:**
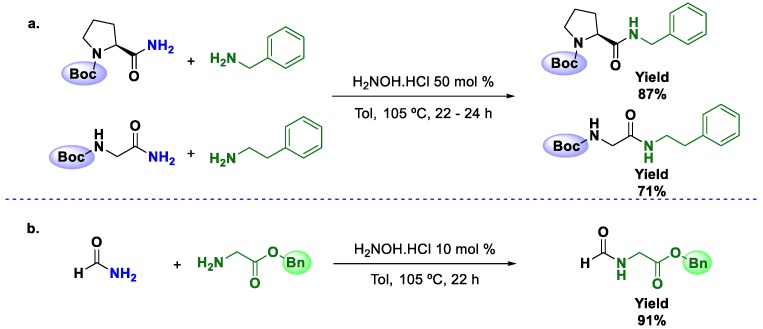
Hydroxylamine hydrochloride as a catalyst of the transamidation reaction of (**a**) substrates with a Boc protecting group and (**b**) α-amino esters with a benzyl group.

The activation of primary amides to promote transamidations using catalytic amounts of hydroxylamine was reported by Williams and coworkers [44]. They screened different catalysts and found that the hydroxylamine salt produced the best results. The study, performed in order to compare the differences between base-free and acid salts, demonstrated that the use of salts has a positive effect on the conversion of primary amides into secondary amides. The optimal conditions for this transformation were toluene at a temperature of 105 °C and the amount of NH_2_OH·HCl varied according to the substrate. The reaction was successful with a wide range of functional groups, including halogens, alkenes, alkynes, free phenolic hydroxyl groups, and heterocycles. Other important observations in the application of this methodology were that the Boc protecting group was unaffected under the typical reaction conditions (Scheme 12a) and that amino acid esters can be *N*-acylated without loss of the ester functionality (Scheme 12b). The highest conversion was generally seen with aliphatic amides; in some cases, only 10 mol % of NH_2_OH·HCl was necessary to reach full conversion in short reaction times. However, the transamidation of secondary amides using this methodology is not effective. 

Hypervalent iodine compounds have undergone impressive developments, and their uses are increasing in many applications in organic synthesis. This progress served as inspiration to Singh and coworkers [45] to propose the first iodine (III)-catalyzed transamidation reaction. The best results were obtained when the reaction was done in the presence of 5 mol % of (diacetoxyiodo)benzene (DIB) under microwave heating (60–150 °C) without any solvent. Gratifyingly, this methodology showed a very good substrate scope with good to excellent yields. A careful analysis of the crude reaction mixtures was performed since the catalyst used is an oxidant and products derived from Hoffman rearrangements were observed. In addition, the use of stoichiometric amounts of catalyst promoted nitrene formation and increased the number of byproducts, but by using catalytic amounts, the transamidation reaction was favored. 

As mentioned earlier in this manuscript, the use of organocatalysts has also been described in transamidation reactions. The study reported by Adimurthy [23], where the process was carried out in the presence of l-proline as an inexpensive catalyst (10 mol %) under solvent-free conditions, suffers from some weaknesses, principally the need for the temperature to be higher than 80 °C and the complete inactivity other amino acid catalysts.

**Scheme 13:**
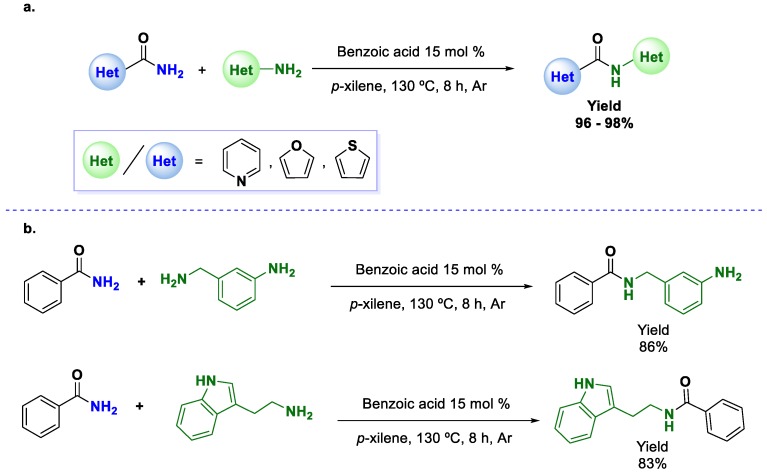
(**a**) Benzoic acid as a catalyst of the transamidation reactions of heterocyclic amides and heterocyclic amines. (**b**) Selectivity of the transamidation reaction using benzoic acid as a catalyst.

Continuing with the revision of inexpensive catalysts in transamidation reactions, benzoic acid was recently used by Xu and coworkers [46], showing very good activity in transamidations. The reaction, performed in xylene at 130 °C, showed very good functional group tolerance, and many substrates demonstrated good reactivity. Among them, the use of heterocyclic amides (nicotinamide, furan-2- and thiophene-2-carboxamide) with heterocyclic amines should be noted (see Scheme 13a). Unfortunately, this method is not effective with aromatic amines, with which it only afforded moderate yields. The main advantages of this methodology are the availability of the catalysts and the excellent selectivity observed. The authors performed a couple of experiments using mixtures of benzamide with 3-aminobenzylamine and benzamide with tryptamine, showing that only one product was formed in each case and thus making this a potential method for the protection of primary amines (Scheme 13b).

**Scheme 14:**
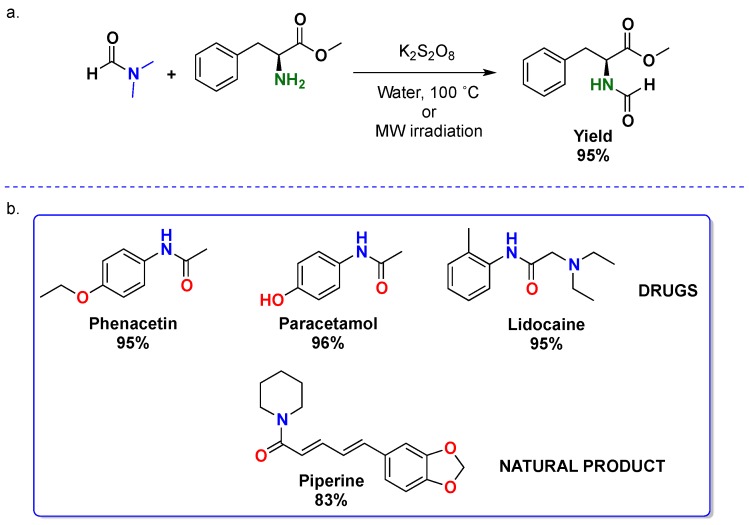
(**a**) *N*-Formylation of α-amino esters promoted by K_2_S_2_O_8_. (**b**) Natural products and drugs obtained by transamidation reactions using K_2_S_2_O_8_.

In addition to these metal-free strategies for transamidation, the use of K_2_S_2_O_8_ in aqueous media was recently described [47]. The reaction can be performed under the influence of microwaves or conventional heating. The study included the screening of other peroxides, such as H_2_O_2_, TBHP, *m*CPBA and oxone, finding that K_2_S_2_O_8_ was the best reactant for the transamidation reaction. Unfortunately, stoichiometric amounts of the promoter are required to achieve complete conversions. This method was applied successfully to L-phenylalanine methyl ester hydrochloride with DMF as the formylating reagent, obtaining the *N*-formyl amino acid ester in 95% yield without any change in the configuration and optical purity, making this method possibly advantageous with chiral substrates (Scheme 14a). The authors showed an application in the synthesis of some very important molecules, such as phenacetin, paracetamol, lidocaine and piperine (Scheme 14b).

Recently, Das and coworkers [48] developed a new H_2_SO_4_-SiO_2_ catalytic system for the direct transamidation of carboxamides, describing a new methodology which complements those previously outlined in this article. Due to the easier ability to manipulate the catalysts, it is described as ecofriendly and low cost. The reaction is performed using 5 mol % H_2_SO_4_-SiO_2_ at 70 °C under solvent-free conditions, and the products were obtained without any purification.

**Scheme 15:**
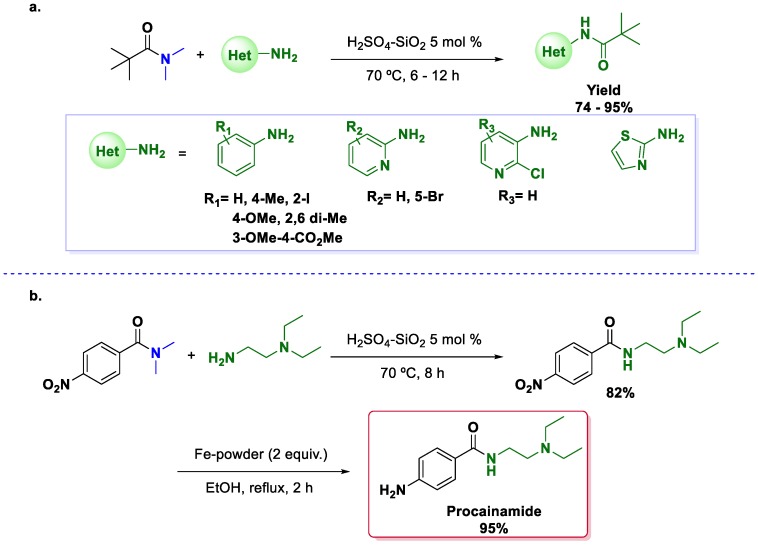
(**a**) Synthesis of *N*-aryl/heteroaryl pivalamides via transamidation catalyzed by H_2_SO_4_-SiO_2_. (**b**) Synthesis of procainamide.

The catalytic system was explored using tertiary amides with substituted aromatic, heteroaromatic, and aliphatic/alicyclic primary amines and some secondary amines as well. The most important application of this methodology was in the synthesis of *N*-aryl/heteroaryl pivalamides, which are an important kind of compound in organic synthesis due to their use as directing groups in many transition-metal catalyzed reactions (Scheme 15a). Furthermore, following this optimized transamidation protocol, the authors reported the synthesis of procainamide, a drug used for the treatment of cardiac arrhythmias (Scheme 15c).

The examples cited in this section make it clear that the transamidation reaction under metal-free conditions is an attractive alternative because of its environmentally friendly and inexpensive nature. Other less relevant reports for metal-free transamidations are also described in the literature. Among them, new catalytic systems using ionic liquids [49], OSU-6 (a modified silica) [50], Fe_3_O_4_-guanidine acetic acid nanoparticles [51], chitosan [52], graphene oxide [53] and nanosized zeolite beta [54] have shown good results and a good substrate scope. However, they do not have any special consideration that make a more detailed discussion necessary.

## 5. Conclusions

The amide moiety is one of the most important functional groups in organic, pharmaceutical and biological chemistry. Its synthesis has been the focus of many researchers; alternative methods to obtain amides are still needed, and this field is in continuous growth. Currently, alternative activation methods for amides are among the most innovative and recent methods; however, the simple activation of the C–N bond in amides by coordination with metals or by interaction with small molecules is an important alternative in amide bond synthesis. Herein, we surveyed the direct activation of amides in transamidation reactions and presented selected examples in this field. We hope this overview will not only that help researchers and serve as inspiration in their future work, but also that they will find it useful in understanding some recently described transamidation processes.
